# Mechanisms influencing transient cytoplasmic protein targeting to intracellular lipid droplets

**DOI:** 10.1042/BST20250461

**Published:** 2026-06-29

**Authors:** Jay L. Laws, Ebony A. Monson, Lachlan A. Wallace, Karla J. Helbig, Cameron J. Reddington

**Affiliations:** 1 Department of Microbiology, Anatomy, Physiology and Pharmacology, La Trobe University, Melbourne, Australia; 2 La Trobe Institute for Molecular Science, La Trobe University, Melbourne, Australia

**Keywords:** CYTOLD, Lipid droplets, Membrane targeting, Post-translational modifications

## Abstract

Lipid droplets (LDs) have a multitude of functions ranging from lipid storage to fighting infection and are decorated with a variety of proteins on their surface that determine their functions and behaviours. Mass spectrometric analysis has identified the vast array of LD-localised proteins, which have recently been shown to be dynamic, changing in response to cellular stress, infection, and altered homeostasis. Here, we review the key mechanisms of cytoplasmic protein interactions with the LD, highlighting conventional features like amphipathic helices, atypical sequence-based motifs, protein–protein interactions, and post-translational modifications that confer dynamic targeting of proteins to the surface of the LD. A better understanding of the transient LD proteome and the mechanisms that confer LD protein targeting will allow researchers to develop a more thorough understanding of LD biology, and the role of LDs in cellular homeostasis and disease.

## Introduction

Lipid droplets (LDs) are evolutionarily conserved, ubiquitous organelles that are comprised of a phospholipid monolayer and neutral lipid core [1]. They have essential roles in regulating diverse cellular processes including lipid metabolism [2], protection against endoplasmic reticulum (ER) stress [3,4], and protection from lipotoxicity [5]. LDs also have roles in cancer, infection and disease (reviewed in [6]), including acting as a fuel source and platform for pathogen replication [7–12], and regulating host immunity following infection [13–16].

The biogenesis of LDs in eukaryotes has been extensively described (reviewed in [17,18]). Typically, LD biogenesis begins with the accumulation of neutral lipids into the ER lumen, causing their aggregation in the inner leaflet. Once sufficient neutral lipids have accumulated within the lumen, the LD begins budding towards the cytosol, with proteins bound to the ER surface transferred onto the nascent LD to become LD resident proteins (Class I proteins, ER to LD or endoplasmic reticulum-to-lipid droplet (ERTOLD), reviewed in [19–21]). Following budding from the ER, it is believed that the proteome of LDs can continually evolve, where proteins from the cytosol can subsequently associate with the LD surface (Class II proteins, cytoplasm to LD or cytoplasm-to-lipid droplet (CYTOLD), reviewed in [22,23]).

At the time of the present mini-review, nearly 40 different mammalian LD proteomes have been published, describing the heterogeneous nature of the LD proteome from numerous organisms and cell types (reviewed in [24,25]). Advancements in mass spectrometry have further expanded our understanding of the diversity of the LD proteome, with several studies describing proteome changes that occur during altered cellular homeostasis, including in hepatic steatosis (fatty liver disease [26,27]), obesity [28], fasting [29], and infection [15,16,30–32]).

Seemingly, all of the above conditions result in significant alterations to the LD proteome. For instance, LDs isolated from mice with hepatic steatosis show increased numbers of ER-associated proteins that are involved in fatty acid catabolism, and decreased association with proteins related to glucose metabolism [26]. The most dramatic changes to the LD proteome have been observed following pathogen infection, where proteins that are involved in lipid metabolism, lipid storage, and immunity are recruited to LDs following bacterial and viral infection [15,16,30–32]. Furthermore, agonists that activate early innate immune cascades, such as lipopolysaccharide and synthetic dsRNA (polyinosinic:polycytidylic acid, poly I:C), stimulate changes to the LD proteome towards enrichment of non-classical LD proteins that have roles in innate immunity [15,16].

In summary, the LD proteome is dynamic, changes rapidly, and contributes to the maintenance of cellular homeostasis. The process of proteins being targeted from the ER membrane to LDs has been described in depth elsewhere [19–21]. The present mini-review will instead focus upon the mechanisms of protein–LD targeting that occur from the cytosol, highlighting key factors in CYTOLD protein localisation that allow the LD proteome to be dynamically regulated. We also highlight aspects of this process that warrant further investigation to deepen our understanding of how the LD proteome recruits such a diverse array of proteins, which may ultimately enable targeted manipulation of this proteome.

## Mechanisms of CYTOLD protein targeting

**Figure 1:**
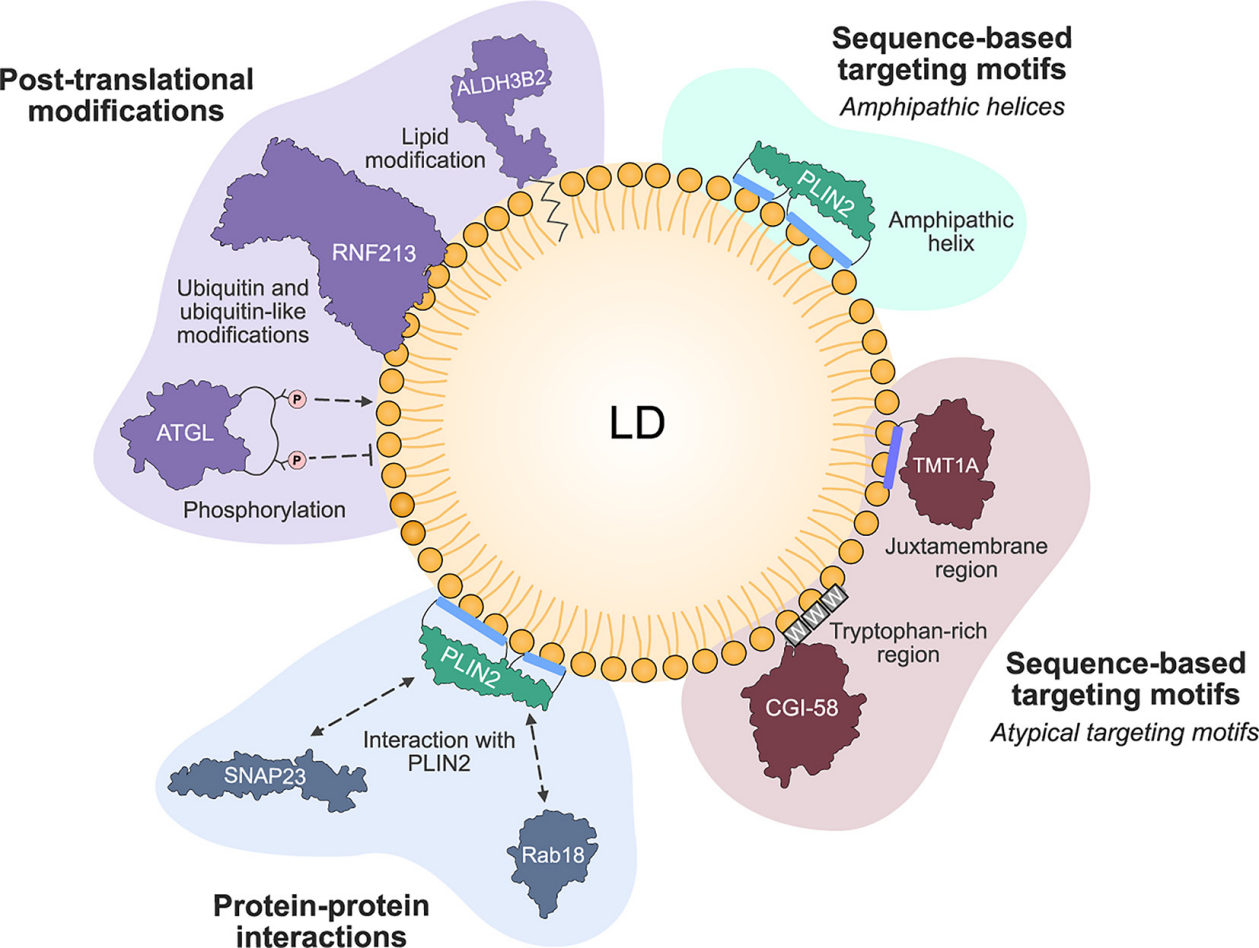
Conventional modes of cytosolic protein localisation with LDs Proteins are shown to be targeted from the cytosol to LDs by sequence-based targeting motifs, protein–protein interactions, and post-translational modifications (bold). Sequence-based targeting motifs are separated into amphipathic helices and atypical targeting motifs. The amphipathic helix of perilipin 2 (PLIN2, aqua) is shown binding to the surface of the LD [34]. Atypical sequence-based motifs (red) are shown to target comparative gene identification-58 (CGI-58) and thiolmethyltransferase 1A (TMT1A) to LDs, via tryptophan-rich and juxtamembrane regions, respectively [35,36]. Protein–protein interactions facilitate a subset of LD binding (blue), where synaptosome-associated protein 23 (SNAP23) and Ras-associated protein found in brain 18 (Rab18) are shown to be recruited to LDs through interaction with PLIN2 [37,38]. Finally, PTMs are shown to regulate LD protein association (purple), where the phosphorylation state of murine adipose triglyceride lipase (ATGL) regulates LD binding, the activities of RING finger protein 213 (RNF213) regulate CYTOLD protein localisation via ubiquitin and ubiquitin-like modifications [39], and modification of aldehyde dehydrogenase 3 family member B2 (ALDH3B2) with lipids facilitates LD targeting [40,41]. The orientation and scale of all depicted proteins in this figure relative to the LD membrane are representative and not based upon specific structural details of protein-LD association.

The various known mechanisms that confer localisation of CYTOLD proteins are outlined in the following sections, where they are grouped into sequence-based targeting motifs, protein–protein interactions, and post-translational modifications (PTMs, Figure 1). Importantly, these features are not unique to LD-localised proteins, as many cytosolic proteins utilise similar mechanisms to target other intracellular membranous organelles. Rather, specificity for LD targeting arises through interplay between these features and the distinctive characteristics of the LD monolayer membrane [19,23,33]. The present mini-review highlights key mechanisms underlying CYTOLD protein localisation, provides representative examples and describes the dynamic regulation of these processes.

## Sequence-based targeting motifs

### Amphipathic helices

Most commonly, cytosolic proteins are targeted to LDs through amphipathic helices, ⍺-helical structures that possess a non-polar face for interaction with phospholipid membranes [34,42]. Amphipathic helices are not unique to LD-localised proteins and can be found in a wide range of proteins that indiscriminately bind membranous structures throughout the cell. Several amphipathic helices can, however, preferentially bind the monolayer surface of the LD [34,42,43]. The length, hydrophobicity and charge of amphipathic helices—and specific properties of the LD membrane—specify LD protein targeting, rather than binding to bilayer membranes on other organelles [44–46]. The binding of proteins that contain amphipathic helices can also be correlated to the types of lipids that the LD has packaged. For instance, perilipin (PLIN) proteins, which are the most well described family of LD-localised proteins, contain amphipathic helix motifs that are preferentially targeted towards triacylglyceride (TAG)- or cholesterol ester-rich LDs (Figure 1, [46,47]). Often, amphipathic helices are encoded as intrinsically disordered regions (IDRs), unstructured regions that assemble into amphipathic helices only upon contact with phospholipid membranes [48,49]. PLIN proteins further exemplify IDR function in the context of the LD, where a disordered 11-mer amino acid repeat region and perilipin/ADRP/TIP47 (PAT) domain fold into amphipathic helices following their association with LDs [50,51]. PTMs have also been described to modulate IDR structure, transiently influencing protein localisation [52–55]. For instance, phosphorylation of the non-receptor kinase cellular sarcoma reduces binding of its N-terminal IDR to the plasma membrane [56]. Further studies should identify specific examples in which amphipathic helix-driven LD localisation is regulated by PTMs and determine how changes to PTM status influence the subcellular localisation of these CYTOLD proteins.

### Atypical targeting motifs

While amphipathic helices are the most described structural feature contributing to LD protein targeting, several other, atypical sequence-based motifs have been shown to mediate localisation of cytosolic proteins to the LD. For instance, CGI-58 relies upon an N-terminal tryptophan-rich region for LD localisation [35]. Furthermore, a hydrophobic motif named the juxtamembrane region has been identified to target TMT1A to LDs; and is a motif also shared by associated with lipid droplets protein 1 (ALDI) and cytochrome b5 reductase 3 (CYB5R3) [36]. Fusion of these juxtamembrane regions to a mutant, constitutively-cysosolic form of Ras-related protein in brain 5 (Rab5) induced Rab5-LD localisation [36]. Similar juxtamembrane regions from asparagine-linked glycosylation 5 homolog (ALG5) and cytochrome P450 family 2 subfamily C member 9 (CYP2C9) failed to target the cytosolic form of Rab5 to LDs as fusion proteins, suggesting that there are other yet to be described mechanisms conferring LD localisation by this motif (Figure 1, [36]). Despite these findings, no ubiquitous LD targeting motif or domain has been identified across the broad spectrum of proteins that are targeted towards the LD membrane.

## Protein–protein interactions

Many proteins that lack known LD targeting sequences often depend upon protein–protein interactions with already anchored proteins to coordinate their localisation to the LD (termed ‘indirect to LD’, indirect-to-lipid droplet (INTOLD) proteins by [22]). For example, PLIN2 recruits several interacting partners to LDs, including SNAP23 [38] and Rab18 (Figure 1, [37]). The functions of these interacting proteins are essential to LD biology, where SNAP23 is crucial for inhibiting cellular glucose uptake [38] and Rab18 regulates TAG content and LD size [37]. Another example is given by the valosin-containing protein (VCP/p97) segregase, which is recruited to LDs through interaction with ubiquitin regulatory X D8 (UBXD8), an ERTOLD protein. Following localisation, VCP regulates the turnover and activity of LD-associated proteins, influencing LD size and lipid metabolism [57]. Notably, the proportion of LD-associated proteins that lack obvious targeting sequences, yet still localise to LDs via protein–protein interactions, remains unknown.

As this research area expands, it will be critical to also understand the role that PTMs may play in regulating these protein interactions. The binding affinities of protein–protein interactions can be regulated by PTMs, where the electrostatic and structural properties of protein interaction sites can be altered [58]. Combined analysis of protein-interaction networks on the LD surface, and the regulatory role of PTMs within these interactions, should be performed to uncover specific examples of how LD protein localisation is dynamically regulated in the context of protein–protein interactions.

## Post-translational modifications

As introduced in the previous sections, PTMs are important to the dynamic regulation of LD protein targeting in many examples to date, driving changes to the LD proteome in response to cellular stimuli. While it is also known that PTMs can drive the localisation of proteins to distinct organelle membranes within a cell [59–62], no studies have yet performed deep analysis of the PTMs present on LD-resident proteins. It has, however, been established that various enzymes required for the attachment and removal of regulatory PTMs are present within the LD proteome. The following sections subsequently describe the role of common PTMs in CYTOLD protein localisation, and relevant enzymes that are targeted towards LDs.

### Phosphorylation

Phosphorylation is one of the most abundant PTMs in mammalian cells and regulates an array of cellular processes, including protein signalling, trafficking, and localisation [63–65]. Various protein kinases are localised to LDs, conferring phosphorylation of resident proteins. For instance, choline kinase alpha 2 (CHKα2) is sequestered to LDs through interaction with PLIN2 and PLIN3. PLIN2/3 subsequently become targeted for phosphorylation by CHKα2 and dissociate from the LD, allowing access by lipases for lipolysis [66]. In another example, murine ATGL is phosphorylated at Ser^406^ by protein kinase A (PKA) and AMP-activated protein kinase (AMPK) to drive LD localisation [67–69]. Conversely, phosphorylation of murine ATGL at Thr^372^ limits LD binding [40]. Although these experiments were performed on murine ATGL, the positions of phosphorylated residues are conserved in humans, suggesting that the localisation of human ATGL may be regulated similarly (Figure 1). It is likely that many other examples of differential phosphorylation exist to coordinate LD protein localisation. Thus, changes to the LD phosphoproteome should be studied in the coming years, to probe the role of phosphorylation in regulating the presence or absence of LD resident proteins.

### Ubiquitin and ubiquitin-like modifications

The modification of proteins by ubiquitin has emerged as a PTM that can regulate virtually all aspects of cellular function. Ubiquitination occurs through a multi-step process, where a 76 amino acid-long protein named ubiquitin is attached to proteins through the sequential activities of a ubiquitin-activating (E1), -conjugating (E2), and -ligating (E3) protein [70–72]. In parallel, there are numerous ubiquitin-like proteins, which share structural similarities to ubiquitin and are covalently attached to protein substrates through a comparable cascade of enzymes. Interferon-stimulated gene 15 (ISG15) and small ubiquitin-related modifier (SUMO) are the ubiquitin-like modifications that are most relevant to association of proteins with the LD surface [16,73–75]. The enzymes that confer attachment of ubiquitin and ubiquitin-like proteins are highly-enriched at LDs, suggesting that the LD may act as a hub for regulation of LD resident proteins through these modifications [16].

Classically, the functions of ubiquitin have been explored in the context of the proteasomal degradation of protein substrates, regulating the presence or absence of proteins at LDs [72,76]. It is now being recognised that ubiquitin can additionally perform non-proteolytic regulatory functions, which may alternatively regulate LD protein localisation [77]. Proteins can be modified either with a single ubiquitin moiety or multiple ubiquitin proteins as a poly-ubiquitin chain, attached through different lysine linkages (either K6, K11, K27, K29, K33, K48, or K63 [78,79]). These lysine linkages confer different functionalities, including protein localisation, protein interactions, and enzymatic activity [78,80]. The ubiquitinome of LD proteins has not yet been studied, and future work should investigate which LD resident proteins are modified with ubiquitin, the types of ubiquitin chains that are attached, changes to localisation that occur following protein ubiquitination, and how the LD ubiquitinome changes following cellular stimulation.

ISG15 is a ubiquitin-like protein that is attached to interferon-stimulated proteins during the immune response (named protein ISGylation, reviewed in [81]). Importantly, an E3 ubiquitin ligase named RING finger protein 213 (RNF213, also known as mysterin) has been identified as a sensor of protein ISGylation in infected cells [39]. RNF213 exerts broad antimicrobial activity—acting as an anti-viral signalling hub to coordinate the immune response towards Gram-positive and Gram-negative bacteria, eukaryotic parasites, and both RNA and DNA viruses [82]—and is significantly enriched at LDs following infection [16,39]. Notably, RNF213 can bind to ISGylated proteins and enrich a broad complement of interferon-stimulated proteins to LDs [39]. RNF213 subsequently appears to be responsible for many of the changes to the LD proteome that occur following infection. Several LD resident proteins are also modified with SUMO, a ubiquitin-like protein that exists in four isoforms in humans (SUMO1-4). For instance, the SUMOylation state of PLIN4 has been shown to alter its LD localisation in mice [83]. Other examples of how LD protein targeting is regulated by SUMO should further be studied to determine how this modification can specifically contribute to the dynamic regulation of the LD proteome.

Recently, it has been shown that ubiquitin can additionally be attached to non-proteinaceous substrates; not only to the protein substrates that have previously been described (reviewed in [77]). For instance, ubiquitin and other ubiquitin-like proteins can be attached to phospholipid headgroups, specifically at phosphatidylethanolamine (PE) [84], a key component of the LD membrane [85]. In this work, phospholipids were shown to be modified with ubiquitin, ISG15 and neural precursor cell expressed developmentally down-regulated protein 8 (NEDD8) on the surface of endosomes, vacuoles and lysosomes [84]. It is not yet known whether these modifications are attached to phospholipids embedded within the LD membrane, nor whether these modifications have a functional role in LD biology or protein-LD association, but is an evolving area of research interest that should be investigated in the context of protein targeting to the LD surface.

### Lipid modifications

**Figure 2:**
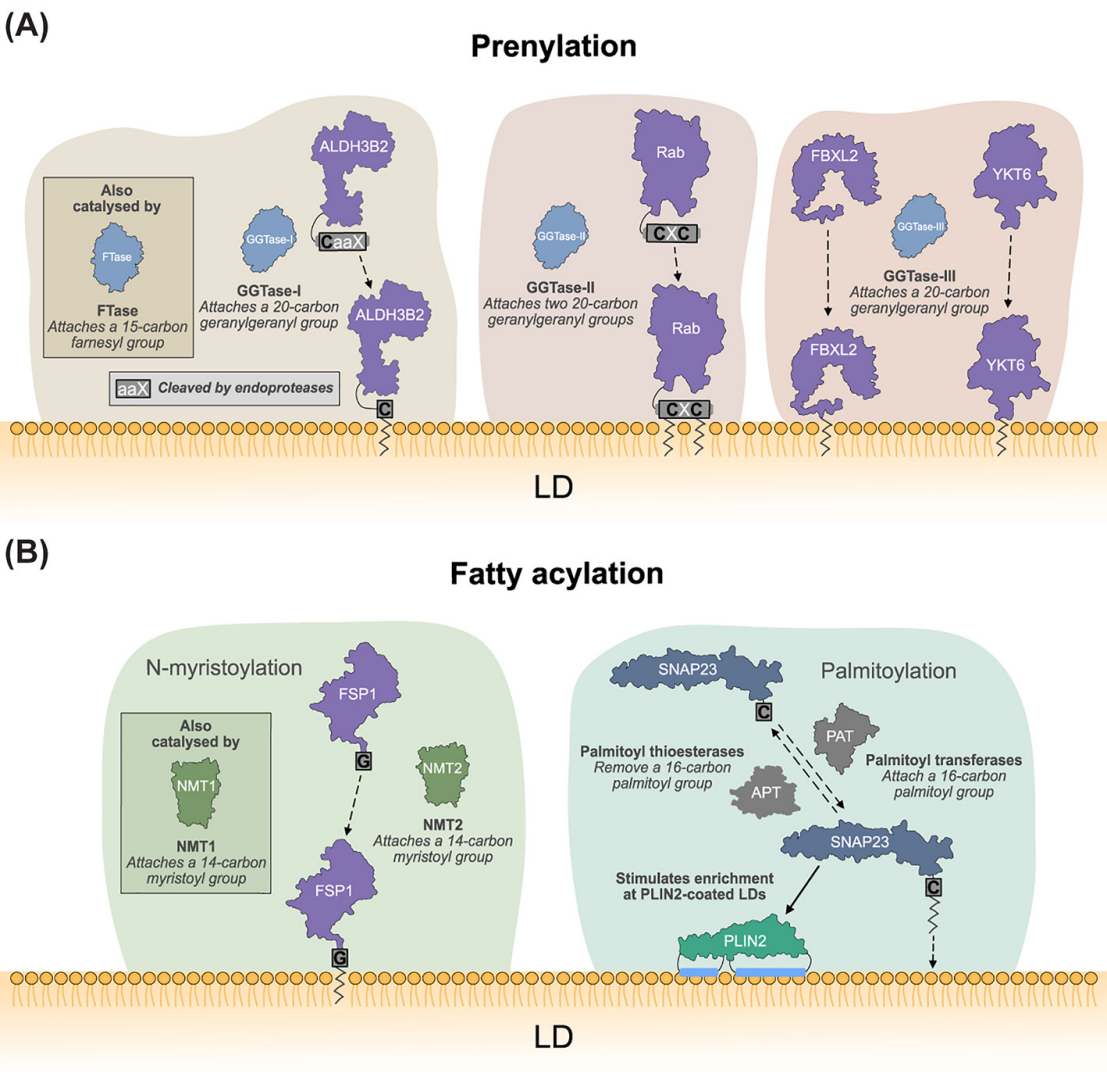
Mechanisms of protein lipid modification Proteins are targeted towards LDs through lipid modification, either by protein prenylation (**A**) or fatty acylation (**B**). (**A**) Enzymes that stimulate protein prenylation are depicted in three groups based on their transfer mechanism. On the left, farnesyl transferase (FTase, inset) and geranylgeranyl transferase I (GGTase-I) are, respectively, shown to attach a 15-carbon farnesyl or 20-carbon geranylgeranyl group to cysteine (C) at the C-terminus of target proteins [90]. A ‘CaaX’ motif is targeted for modification, where ‘a’ is an aliphatic amino acid and ‘X’ is any amino acid. The C-terminal residues ‘aaX’ are cleaved by endoproteases (inset). Geranylgeranyl transferase II (GGTase-II) (centre) is shown to attach two 20-carbon geranylgeranyl groups to a pair of cysteine residues (CXC or CCXX motif) at the C-terminus of Ras-related protein found in brain (Rab) GTPases [91,92]. On the right, geranylgeranyl transferase III (GGTase-III) is shown to attach a 20-carbon geranylgeranyl group to two known substrates, F-box and leucine rich repeat protein 2 (FBXL2) and synaptobrevin homolog (YKT6) [93]. (**B**) Fatty acylation involves modification of proteins with either a 14-carbon myristoyl (left) or 16-carbon palmitoyl group (right). On the left, ferroptosis suppressor protein 1 (FSP1) is shown to be N-myristoylated by N-myristoyl transferase 2 (NMT2) at an N-terminal glycine residue. This modification can also be catalysed by N-myristoyl transferase 1 (NMT1, inset) [94,95]. On the right, SNAP23 is shown to be modified with a 16-carbon palmitoyl group by a palmitoyl transferase (PAT) enzyme. Protein palmitoylation is reversible; an acyl-palmitoyl thioesterase (APT) enzyme is shown to remove the palmitoyl group from SNAP23. When palmitoylated, SNAP23 is enriched at PLIN2-coated LDs to stimulate their protein–protein interaction [38] (solid arrow). The orientation and scale of all depicted proteins in this figure relative to the LD membrane are representative and not based upon specific structural details of protein–LD association.

Similarly to the proteinaceous modifications described above, the lipidation of proteins involves covalent attachment of lipids (e.g. fatty acids, isoprenoids, cholesterol) to amino acids, usually at cysteine (reviewed in [86,87]). Generally, attachment of lipid modifications increases protein hydrophobicity [87]. Lipid modifications are often considered necessary, but not sufficient, for the localisation of proteins to membranous organelles, and act in concert with other targeting mechanisms that confer specificity toward the LD surface [88,89]. Prenylation and acylation are the types of lipid modification that are most relevant to LD protein localisation (Figure 2).

#### Prenylation

Prenylation is an irreversible PTM where an isoprenoid group, either a 15-carbon farnesyl group or a 20-carbon geranylgeranyl group, is attached to a conserved cysteine residue near the C-terminus of target proteins (reviewed in [90,96]). Many LD-localised proteins undergo prenylation, which can affect their maturation, localisation, and interaction with the LD membrane [41,93,97,98].

Several different prenyltransferase enzymes catalyse the prenylation of proteins. FTase and GGTase I catalyse attachment of a single farnesyl or geranylgeranyl isoprenoid group, respectively, to cysteine residues [99]. These enzymes recognise a C-terminal CaaX motif, where ‘C’ is a cysteine, ‘a’ is an aliphatic amino acid, and ‘X’ determines which of the two prenylation processes are taking place; farnesylation occurs when ‘X’ is serine, cysteine, glutamine or methionine, and geranylgeranylation occurs when ‘X’ is leucine or isoleucine [99]. Following prenylation, the terminal ‘aaX’ residues are cleaved by endoproteases, producing a protein that is prenylated at the C-terminus (Figure 2A, [96]). Prenylation by FTase and GGTase I confer localisation of target proteins to membranes, including to the LD. For example, geranylgeranylation of the enzyme ALDH3B2 by GGTase I is required for its LD localisation [41].

Another subset of protein prenylation is catalysed by GGTase-II. GGTase-II catalyses the addition of two geranylgeranyl groups to neighbouring cysteine residues in sequences like CXC or CCXX, close to the C-terminus of Rab GTPases [91]. Rab GTPases are commonly localised to LDs to coordinate the transport of lipids for storage and metabolism [92,100]. Alongside prenylation, the localisation of Rab proteins from a predominantly cytosolic pool to a membrane-bound, active species is tightly regulated by interacting proteins, including guanine nucleotide exchange factors and GTPase-activating proteins, which coordinate Rab nucleotide cycling [92,101]. As many as 30 different Rab GTPases have been identified in LD proteomic studies (Figure 2A, reviewed in [92]), although Rab18 is the best-characterised. Rab18 was introduced earlier in the present mini-review as a PLIN2-interacting protein [37], and its localisation to LDs exemplifies the multi-factorial complexity of lipid modification-driven protein targeting, being governed by prenylation, a diverse protein interaction network and other factors [37,92,101–103]. Interestingly, Rab18 is also unusual among Rab GTPases, in containing a C-terminal CAAX motif that undergoes mono-prenylation, rather than a canonical di-prenylation motif [102–104]. Finally, proteins can alternatively be prenylated by GGTase-III. Currently, only two substrates have been identified for GGTase-III; FBXL2 and YKT6, which are localised to LDs following their prenylation, and, respectively, have essential roles in lipid metabolism and membrane organisation [105–108]. Altogether, prenylation is a key contributor to LD protein localisation, acting as a lipid anchor to support the localisation of a diverse array of regulatory proteins [25,109].

#### Fatty acylation

Proteins are modified by fatty acylation through attachment of fatty acids at specific residues. The types of fatty acid chains that are added to target proteins are most commonly myristoyl (14-carbon) or palmitoyl (16-carbon) groups [110]. Like prenylation, fatty acylation enhances protein-membrane association and stimulates protein–protein interactions (reviewed in [88]).

N-myristoylation involves the addition of a 14-carbon unsaturated fatty acid (myristic acid) to N-terminal glycine residues by an NMT enzyme (reviewed in [111]). N-myristoylation drives the localisation of proteins to the LD and can occur co-translationally or as a PTM [112], with humans expressing two NMT enzymes, each having distinct substrate affinities and cellular functions [113]. Both ERTOLD and CYTOLD proteins are N-myristoylated. For instance, ankyrin repeat domain 22 (ANKRD22), a transmembrane ERTOLD protein, is N-myristoylated to assist with targeting during LD biogenesis [112]. A variety of CYTOLD proteins are also N-myristoylated, including FSP1—an enzyme that prevents peroxidation of neutral lipids stored within the LD core—which is N-myristoylated by NMT2 (Figure 2B, [94,95]). However, only a handful of proteins have been studied in the context of LD targeting by N-myristoylation, and it is not yet known what other features may act in concert with the lipid modification to direct these proteins specifically toward the LD membrane, rather than other membranous organelles.

Palmitoylation is the most common PTM in the acylation family and involves addition of a 16-carbon fatty acid, palmitate (palmitic acid), to target proteins by a palmitoyl acyltransferase enzyme (PAT, [114,115]). Notably, protein palmitoylation is reversible, where it can be removed by an APT enzyme, which distinguishes it from the other lipid modifications that we have described [114,115]. Examples of palmitoylation in the context of LDs include modification of SNAP23 to facilitate localisation to PLIN2-coated LDs (Figure 2B, [116]), and palmitoylation of elmo domain containing 2 (ELMOD2), a LD-localised GTPase [117].

Notably, the role of lipid modifications in targeting proteins to LDs and other intracellular membranes has only recently been described. Further technological advancements are likely to assist in identifying the entire complement of LD resident proteins that are modified with lipids to regulate their membrane localisation. It will be interesting to further study how lipid modification states are regulated under basal conditions and change during altered cellular homeostasis.

## Conclusions

In the present review, we highlight the extensive diversity and complexity of the mechanisms that underlie cytosolic protein interactions with the LD. Current evidence shows that cytosolic proteins can associate with LDs through sequence-defined structural features, protein–protein interactions, and PTMs, which are each regulated to confer dynamic changes to the LD proteome. However, scientific advancement is constantly growing our understanding of the breadth of proteins able to be targeted to the LD; making it likely that several other, undescribed modes of LD protein localisation may exist alongside those discussed in the present mini-review. Future multidisciplinary studies are likely to further our knowledge of how cytoplasmic proteins are targeted toward LDs, clarifying the mechanisms that favour their localisation to the LD monolayer membrane. Various experimental strategies have been highlighted in the present mini-review, which emphasize the need to determine a *bona fide* transient LD proteome across different physiological conditions. Once the complement of CYTOLD proteins has been established, researchers should aim to further expand upon our understanding of the mechanisms that facilitate CYTOLD protein targeting, potentially by determining the full extent of how these proteins interact with one another, or are modulated by PTMs, on the surface of LDs. Together, these findings will be critical to unravelling the regulatory functions of LDs in cells and may ultimately guide new therapeutic approaches for diseases marked by dysregulated protein-LD targeting.

## Perspectives

- Proteins are dynamically targeted towards LDs from the cytoplasm, allowing the LD proteome to adapt in response to cellular stress, infection, and altered homeostasis. Recent advances have substantially expanded the known breadth of the transient LD proteome, highlighting the need to better understand how proteins are selectively targeted from the cytoplasm to the LD.
- Emerging evidence suggests that cytoplasmic proteins are targeted towards LDs by embedded structural features, including amphipathic helices, IDRs and atypical sequence-based motifs, which function alongside protein–protein interactions and PTMs to coordinate LD protein localisation.
- The mechanisms that transiently regulate the targeting of proteins from the cytoplasm to LDs are not yet fully understood, and resolving the *bona fide* transient LD proteome across diverse physiological conditions will be essential to further identify unifying principles that govern cytosolic LD targeting. Progress towards a more mechanistic understanding of these processes could be advanced through several complementary approaches, including: (1) dissecting the features of individual targeting mechanisms that confer specificity toward the LD surface; (2) mapping the interaction networks of INTOLD proteins; and (3) constructing an atlas of post-translational modifications present on transient LD proteins across different physiological cellular states.

## Open Access

Open access for this article was enabled by the participation of La Trobe University in an all-inclusive Read & Publish agreement with Portland Press and the Biochemical Society under an agreement with CAUL.

## Abbreviations

ALDH3B2: aldehyde dehydrogenase 3 family member B2
ALDI: associated with lipid droplets protein 1
ALG5: asparagine-linked glycosylation 5 homolog
AMPK: AMP-activated protein kinase
ANKRD22: ankyrin repeat domain 22
APT: acyl-palmitoyl thioesterase
ATGL: adipose triglyceride lipase
CGI-58: comparative gene identification-58
CHKα2: choline kinase alpha 2
CYTOLD: cytoplasm-to-lipid droplet
E1: E1 ubiquitin-activating enzyme
E2: E2 ubiquitin-conjugating enzyme
E3: E3 ubiquitin-ligating enzyme
ELMOD2: elmo domain containing 2
ER: endoplasmic reticulum
ERTOLD: endoplasmic reticulum-to-lipid droplet
FBXL2: F-box and leucine rich repeat protein 2
FSP1: ferroptosis suppressor protein 1
FTase: farnesyl transferase
GGTase-I–III: geranylgeranyl transferase I–III
IDR: intrinsically disordered region
INTOLD: indirect-to-lipid droplet
ISG15: interferon-stimulated gene 15
LD: lipid droplet
NEDD8: neural precursor cell expressed developmentally down-regulated protein 8
NMT1/2: N-myristoyl transferase 1/2
PAT domain: perilipin/ADRP/TIP47 domain
PAT enzyme: palmitoyl transferase enzyme
PE: phosphatidylethanolamine
Poly I:C: polyinosinic:polycytidylic acid
PKA: protein kinase A
PLIN2: perilipin 2
PTM: post-translational modification
Rab: Ras-associated protein found in brain
Rab18: Ras-associated protein found in brain 18
Rab5: Ras-related protein in brain 5
RNF213: RING finger protein 213
SNAP23: synaptosome-associated protein 23
SUMO1–4: small ubiquitin-related modifier 1–4
TAG: triacylglyceride
TMT1A: thiolmethyltransferase 1A
UBXD8: ubiquitin regulatory X D8
VCP: valosin-containing protein
YKT6: synaptobrevin homolog
